# RNU4ATAC-opathy: Clinical, molecular and transcriptomic insights from a large cohort

**DOI:** 10.1016/j.gim.2026.102633

**Published:** 2026-06-19

**Authors:** Dena R. Matalon, Angela L. Duker, Taylor M. Arriaga, Kathryn Russell, Hector Rodrigo Mendez, Devon E. Bonner, Margaret E. Harley, Moriel Singer-Berk, Monica H. Wojcik, Lynn Pais, Stephanie DiTroia, Melanie O’Leary, Thomas Cassini, Kimberly Ezell, Anne D. Niehaus, Julie Kaplan, David S. Wargowski, Cory J. Smid, Emily D. Longenecker, Ana Maria Rodriguez Barreto, Danny E. Miller, Alexandra C. Keefe, Laurel Calderwood, Gregory M. Enns, Mustafa Tekin, Stephanie A. Bivona, Neeta L. Vora, Kelly L. Gilmore, Tahir N. Khan, Erica E. Davis, Amber W Wang, Sameena Khan, Sateesh Maddirevula, Lama AlAbdi, Omar Abuyousef, Hanan E. Shamseldin, Salwa Alkhalifi, Firdous Abdulwahab, Mashael Alqahtani, Zainab A. Alhumaidi, Seba Nadeef, Amal M. Al Hashem, Khadijah Bakur, Eissa A. Faqeih, Ebtesam Abdalla, Angus Clarke, Elaine Fletcher, Wee Teik Keng, Lilian Bomme Ousager, Deepthi C. de Silva, Muzhirah Haniffa, Francesca Mari, Wayne Lam, Jennifer Campbell, Tessa Homfray, Sheela Nampoothiri, Chumei Li, Bimal P. Chaudhari, Kristen Truxal, Members of the Genomics Research to Elucidate the Genetics of Rare Diseases (GREGoR) consortium:, Siwaar Abouhala, Kaileigh Ahlquist, Miguel Almalvez, Emily Alsentzer, Raquel Alvarez, Mutaz Amin, Kailyn Anderson, Peter E. Anderson, Euan Ashley, Themistocles Assimes, Light Auriga, Christina Austin-Tse, Michael J. Bamshad, Rebekah Barrick, Rebekah Barrick, Samantha Baxter, Sairam Behera, Shaghayegh Beheshti, Gill Bejerano, Sami Belhadj, Seth Berger, Jon Bernstein, Sabrina Best, Benjamin Blankenmeister, Elizabeth E. Blue, Krista Bluske, Eric Boerwinkle, Emily Bonkowski, Devon Bonner, Philip M. Boone, Philip M. Boone, Leandros Boukas, Denver Bradley, Harrison Brand, Kati J. Buckingham, Daniel Calame, Colleen Carlston, Jennefer Carter, Silvia Casadei, Lisa Chadwick, Clarisa Chavez, Ziwei Chen, Yong-Han Cheng, Ivan Chinn, Jessica X. Chong, Zeynep Coban-Akdemir, Andrea J. Cohen, Sarah Conner, Matthew P. Conomos, Karen Coveler, Ya Allen Cui, Zain Dardas, Colleen P. Davis, Moez Dawood, Ivan de Dios, Celine de Esch, Celine de Esch, Emmanuèle Délot, Salil Deshpande, Stephanie DiTroia, Harsha Doddapaneni, Haowei Du, Michael Duyzend, Michael Duyzend, Michael Duyzend, Iman Egab, Evan E. Eichler, Sara Emami, Ivy Evergreen, Jamie Fraser, Vincent Fusaro, Mira Gandhi, Vijay Ganesh, Brandon Garcia, Kiran Garimella, Richard Gibbs, Sophia B. Gibson, Casey Gifford, Carmen Glaze, Pagé Goddard, Stephanie Gogarten, Nikhita Gogate, William W. Gordon, John E. Gorzynski, William Greenleaf, Christopher Grochowski, Emily Groopman, Emily Groopman, Rodrigo Guarischi-Sousa, Sanna Gudmundsson, Jonas A. Gustafson, Stacey Hall, Caitlin Harrington, John Harting, William T. Harvey, Sohaib Hassan, Megan Hawley, Benjamin D. Heavner, Martha Horike-Pyne, Yun-Hua Hsiao, Jianhong Hu, Yongqing Huang, Karan Jaisingh, Minal Jamsandekar, Gail P. Jarvik, Tanner Jensen, Shalini Jhangiani, David Jimenez-Morales, Christopher Jin, Aimee Juan, Ahmed K. Saad, Jessica Kain, Rachid Karam, Laura Keehan, Rupesh Kesharwani, Charles Hadley King, Julia Klugherz, Arthur Ko, Anshul Kundaje, Soumya Kundu, Samuel M. Lancaster, Katie Larsson, Arthur Lee, Gabrielle Lemire, Jesse Levine, Richard Lewis, Wei Li, Yidan Li, Pengfei Liu, Bojan Losic, Jonathan LoTempio, James Jim Lupski, Jialan Ma, Daniel MacArthur, Annelise Y. Mah-Som, Medhat Mahmoud, Brian Mangilog, Dana Marafi, Sofia Marmolejos, Daniel Marten, Eva Martinez, Colby T. Marvin, Shruti Marwaha, F. Kumara Mastrorosa, Dena Matalon, Taylor Maurer, Susanne May, Sean R. McGee, Lauren Meador, Heather C. Mefford, Hector Rodrigo Mendez, Olfa Messaoud, Alexander Miller, Danny E. Miller, Stephen Montgomery, Yulia Mostovoy, Mariana Moyses, Mariana Moyses, Chloe Munderloh, Donna Muzny, Ashana Neale, Sarah C. Nelson, Matthew B. Neu, Thuy-mi P. Nguyen, Jonathan Nguyen, Robert Nussbaum, Emily O'Heir, Melanie O'Leary, Briana O'Leary, Sebastian Ochoa Gonzalez, Jeren Olsen, Ikeoluwa Osei-Owusu, Anne OÄôDonnell-Luria, Miranda P.G. Zalusky, Evin Padhi, Lynn Pais, Piyush Panchal, Shruti Pande, Karynne E. Patterson, Sheryl Payne, Davut Pehlivan, Davut Pehlivan, Paul Petrowski, Alicia Pham, Georgia Pitsava, Astaria/Sara Podesta, Elizabeth Porter, Jennifer Posey, Jaime Prosser, Guanghao Qi, Wanqiong Qiao, Thomas Quertermous, Archana Rai, Heidi Rehm, Chloe Reuter, Matthew A. Richardson, Andres Rivera-Munoz, Lindsay Romo, Oriane Rubio, Kathryn Russell, Aniko Sabo, Monica Salani, Monica Salani, Kaitlin Samocha, Alba Sanchis-Juan, Sarah Savage, Stuart Scott, Evette Scott, Adriana E. Sedeño-Cortés, Fritz Sedlazeck, Jillian Serrano, Gulalai Shah, Ali Shojaie, Moriel Singer-Berk, Mugdha Singh, Riya Sinha, Joshua D Smith, Kevin Smith, Hana Snow, Michael Snyder, Kayla Socarras, Olivia M. Sommerland, Lea M. Starita, Brigitte Stark, Sarah Stenton, Sarah Stenton, Andrew B. Stergachis, Adrienne Stilp, Suchitra Sudarshan, V. Reid Sutton, V. Reid Sutton, Jui-Cheng Tai, Jui-Cheng Tai, Michael Talkowski, Christina Tise, Catherine C. Tong, Philip Tsao, Rachel Ungar, Grace VanNoy, Eric Vilain, Gaby Villard, Isabella Voutos, Kim Walker, Juliana Walrod, Chia-Lin Wei, Ben Weisburd, Jeffrey M Weiss, Chris Wellington, Ziming Weng, Lauren Westerfield, Emily Westheimer, Marsha Wheeler, Matthew Wheeler, Laurens Wiel, Michael Wilson, Monica Wojcik, Issac Wong, Frank Wong, Quenna Wong, Changrui Xiao, Jiaoyang Xu, Rachita Yadav, Rachita Yadav, Yao Yang, Qian Yi, Jiye Yu, Bo Yuan, Christina Zakarian, Jianhua Zhao, Jimmy Zhen, Members of the Undiagnosed Diseases Network:, Alyssa A. Tran, Arjun Tarakad, Ashok Balasubramanyam, Brendan H. Lee, Carlos A. Bacino, Daryl A. Scott, Elaine Seto, Gary D. Clark, Hongzheng Dai, Hsiao-Tuan Chao, Ivan Chinn, James P. Orengo, Jennifer E. Posey, Jill A. Rosenfeld, Kim Worley, Lindsay C. Burrage, Lisa T. Emrick, Lorraine Potocki, Monika Weisz Hubshman, Richard A. Lewis, Ronit Marom, Seema R. Lalani, Shamika Ketkar, Tiphanie P. Vogel, William J. Craigen, Lauren Blieden, Jared Sninsky, Hugo J. Bellen, Michael F. Wangler, Oguz Kanca, Shinya Yamamoto, Christine M. Eng, Patricia A. Ward, Pengfei Liu, Adeline Vanderver, Cara Skraban, Edward Behrens, Gonench Kilich, Kathleen Sullivan, Kelly Hassey, Ramakrishnan Rajagopalan, Rebecca Ganetzky, Vishnu Cuddapah, Anna Raper, Daniel J. Rader, Giorgio Sirugo, Vaidehi Jobanputra, Allyn McConkie-Rosell, Kelly Schoch, Mohamad Mikati, Nicole M. Walley, Rebecca C. Spillmann, Vandana Shashi, Alan H. Beggs, Calum A. MacRae, David A. Sweetser, Deepak A. Rao, Edwin K. Silverman, Elizabeth L. Fieg, Frances High, Gerard T. Berry, Ingrid A. Holm, J. Carl Pallais, Joan M. Stoler, Joseph Loscalzo, Lance H. Rodan, Laurel A. Cobban, Lauren C. Briere, Matthew Coggins, Melissa Walker, Richard L. Maas, Susan Korrick, Jessica Douglas, Audrey Stephannie C. Maghiro, Cecilia Esteves, Emily Glanton, Isaac S. Kohane, Kimberly LeBlanc, Rachel Mahoney, Shamil R. Sunyaev, Shilpa N. Kobren, Brett H. Graham, Erin Conboy, Francesco Vetrini, Kayla M. Treat, Khurram Liaqat, Lili Mantcheva, Stephanie M. Ware, Breanna Mitchell, Brendan C. Lanpher, Devin Oglesbee, Eric Klee, Filippo Pinto e Vairo, Ian R. Lanza, Kahlen Darr, Lindsay Mulvihill, Lisa Schimmenti, Queenie Tan, Surendra Dasari, Adriana Rebelo, Carson A. Smith, Deborah Barbouth, Guney Bademci, Joanna M. Gonzalez, Kumarie Latchman, LéShon Peart, Mustafa Tekin, Nicholas Borja, Stephan Zuchner, Stephanie Bivona, Willa Thorson, Herman Taylor, Andrea Gropman, Barbara N. Pusey Swerdzewski, Camilo Toro, Colleen E. Wahl, Donna Novacic, Ellen F. Macnamara, John J. Mulvihill, Maria T. Acosta, Precilla D'Souza, Valerie V. Maduro, Ben Afzali, Ben Solomon, Cynthia J. Tifft, David R. Adams, Elizabeth A. Burke, Francis Rossignol, Heidi Wood, Jiayu Fu, Joie Davis, Leoyklang Petcharet, Lynne A. Wolfe, Margaret Delgado, Marie Morimoto, Marla Sabaii, MayChristine V. Malicdan, Neil Hanchard, Orpa Jean-Marie, Wendy Introne, William A. Gahl, Yan Huang, Aimee Allworth, Andrew Stergachis, Danny Miller, Elizabeth Blue, Elizabeth Rosenthal, Elsa Balton, Emily Shelkowitz, Eric Allenspach, Fuki M. Hisama, Gail P. Jarvik, Ghayda Mirzaa, Ian Glass, Kathleen A. Leppig, Katrina Dipple, Mark Wener, Martha Horike-Pyne, Michael Bamshad, Peter Byers, Sam Sheppeard, Sirisak Chanprasert, Virginia Sybert, Wendy Raskind, Nitsuh K. Dargie, Beth A. Martin, Chloe M. Reuter, Devon Bonner, Elijah Kravets, Holly K. Tabor, Jacinda B. Sampson, Jason Hom, Jennefer N. Kohler, Jonathan A. Bernstein, Kevin S. Smith, Matthew T. Wheeler, Meghan C. Halley, Page C. Goddard, Paul G. Fisher, Rachel A. Ungar, Raquel L. Alvarez, Shruti Marwaha, Terra R. Coakley, Euan A. Ashley, Ali Al-Beshri, Anna Hurst, Bruce Korf, Kaitlin Callaway, Martin Rodriguez, Tammi Skelton, Andrew B. Crouse, Jordan Whitlock, Mariko Nakano-Okuno, Matthew Might, William E. Byrd, Changrui Xiao, Eric Vilain, Jose Abdenur, Kathyrn Singh, Rebekah Barrick, Sanaz Attaripour, Suzanne Sandmeyer, Tahseen Mozaffar, Albert R. La Spada, Elizabeth C. Chao, Maija-Rikka Steenari, Alden Huang, Brent L. Fogel, Esteban C. Dell'Angelica, George Carvalho, Julian A. Martínez-Agosto, Manish J. Butte, Martin G. Martin, Naghmeh Dorrani, Neil H. Parker, Rosario I. Corona, Stanley F. Nelson, Yigit Karasozen, Aaron Quinlan, Alistair Ward, Ashley Andrews, Corrine K. Welt, Dave Viskochil, Erin E. Baldwin, John Carey, Justin Alvey, Laura Pace, Lorenzo Botto, Nicola Longo, Paolo Moretti, Rebecca Overbury, Russell Butterfield, Steven Boyden, Thomas J. Nicholas, Matt Velinder, Gabor Marth, Pinar Bayrak-Toydemir, Rong Mao, Monte Westerfield, Brian Corner, John A. Phillips, Kimberly Ezell, Lynette Rives, Rizwan Hamid, Serena Neumann, Ashley McMinn, Joy D. Cogan, Thomas Cassini, Alex Paul, Dana Kiley, Daniel Wegner, Erin McRoy, Jennifer Wambach, Kathy Sisco, Patricia Dickson, F. Sessions Cole, Dustin Baldridge, Jimann Shin, Lilianna Solnica-Krezel, Stephen Pak, Timothy Schedl, Hector Rodrigo Mendez, Brianna Tucker, Beatriz Anguiano, Mia Levanto, Suha Bachir, Laurens Wiel, Stephen B Montgomery, Tanner D Jensen, John E. Gorzynski, Sara Emami, Laura Keehan, Jennifer Schymick, Taylor Maurer, Alexander Miller, Andres Vargas, Amanda M. Shrewsbury, Bianca E. Russell, Layal F. Abi Farraj, Elizabeth A Worthey, Tarun KK Mamidi, Brandon M Wilk, Rachel Li, Jennifer Morgan, Chun-Hung Chan, Paul Berger, Mohamad Saifeddine, Isum Ward, Jason Schend, Megan Bell, Francisco B Velasquez, Taylor Beagle, Miranda Leitheiser, Runjun Kumar, Donald Basel, Michael Muriello, Brett Bordini, Michael Zimmermann, Abdul Elkadri, James Verbsky, Julie McCarrier, Jonathan A. Bernstein, Stephen B. Montgomery, Matthew T. Wheeler, Fowzan S. Alkuraya, Anne O’Donnell-Luria, Andrew P. Jackson, Ian M. Campbell, Vijay S. Ganesh, Nic Robertson, Gabrielle Lemire

**Affiliations:** 1 Division of Medical Genetics, Department of Pediatrics, Stanford University, CA, USA; 2 Division of Orthogenetics, Department of Pediatrics, Nemours Children's Hospital, Wilmington, DE, USA; 3 Department of Genetics, Stanford University, CA, USA; 4 Broad Institute Center for Mendelian Genomics, Program in Medical and Population Genetics, Broad Institute of MIT and Harvard, Cambridge, MA, USA; 5 Department of Medicine, Stanford University, CA, USA; 6 MRC Human Genetics Unit, Institute of Genetics and Cancer, University of Edinburgh, Edinburgh, UK; 7 Division of Genetics and Genomics, Boston Children's Hospital, Boston, MA, USA; Harvard Medical School, Boston, MA, USA; 8 Vanderbilt University Medical Center, Nashville, TN, USA; 9 Cleveland Clinic Foundation, Cleveland, OH, USA; 10 Division of Genetics and Metabolism, Department of Pediatrics, University of Wisconsin School of Medicine and Public Health, Madison, WI, USA; 11 Division of clinical genetics, Nicklaus Children's Hospital, Miami, Fl, USA; 12 Division of Genetic Medicine, Department of Pediatrics; Department of Laboratory Medicine and Pathology; Brotman Baty Institute for Precision Medicine, University of Washington and Seattle Children’s Hospital, Seattle, WA, USA; 13 Division of Genetic Medicine, Department of Pediatrics, University of Washington and Seattle Children’s Hospital, Seattle, WA, USA; 14 John T. Macdonald Foundation Department of Human Genetics, University of Miami Miller School of Medicine, Miami, Fl, USA; 15 Obstetrics and Gynecology; Division of Maternal Fetal Medicine, UNC Chapel Hill School of Medicine, Chapel Hill, NC, USA; 16 Stanley Manne Children's Research Institute, Ann & Robert H. Lurie Children's Hospital of Chicago, Chicago, IL, USA; 17 Department of Pediatrics and Department of Cell and Developmental Biology, Feinberg School of Medicine, Northwestern University, Chicago, IL, USA; 18 ORIC Pharmaceuticals; 19 Department of Translational Genomics, Center for Genomic Medicine, King Faisal Specialist Hospital and Research Center, Riyadh, Saudi Arabia; 20 Precision Medicine Laboratory Department, Center for Genomic Medicine, King Faisal Specialist Hospital and Research Center, Riyadh, Saudi Arabia; College of Medicine, Alfaisal University, Riyadh, Saudi Arabia; 21 Newborn Screening program, Ministry of Health, Eastern Province, Saudi Arabia; 22 Lifera Omics; 23 Section of Medical Genetics, Children's Specialist Hospital, King Fahad Medical City, Riyadh, Saudi Arabia; 24 Human Genetics Department, Medical Research Institute, Alexandria University, Egypt; 25 Division of Cancer and Genetics, School of Medicine, Cardiff University, Cardiff, Wales, UK; 26 Department of Clinical Genetics, Centre for Genomic and Experimental Medicine, Western General Hospital, Crewe Road South, Edinburgh, UK; 27 Department of Genetics, Hospital Kuala Lumpur, Malaysia; 28 Department of Clinical Genetics, Odense University Hospital, Odense, Denmark; Department of Clinical Research, University of Southern Denmark, Odense, Denmark; 29 Department of Physiology, Faculty of Medicine, University of Kelaniya, Sri Lanka; 30 Department of Medicine, Surgery and Neurosciences, University of Siena, Italy and Clinical Pathology Unit, University Hospital of Siena, Italy; 31 South East of Scotland Clinical Genetics Service, Edinburgh, UK; 32 Clinical Genomics Service, Leeds Teaching Hospitals NHS Trust, Leeds, UK; 33 Medical Genetics Service, St George’s University Hospital and Royal Brompton Hospital London, London, UK; 34 Department of Pediatric Genetics, Amrita Institute of Medical Sciences & Research Centre, Kochi, India; 35 Department of Paediatrics, McMaster University Medical Center, Hamilton, ON, Canada; 36 Division of Genetics and Genomic Medicine, Nationwide Children’s Hospital, Columbus, OH, USA; 37 Department of Pediatrics, The Ohio State University, Columbus, OH, USA; 38 Department of Pediatrics, Stanford University School of Medicine, Stanford University, Stanford, CA, USA; 39 Department of Biomedical Data Science, Stanford University, CA, USA; 40 Center for Inherited Cardiovascular Disease, Stanford Medicine, CA, USA; 41 Division of Human Genetics, Department of Pediatrics, Children's Hospital of Philadelphia, Perelman School of Medicine, University of Pennsylvania, Philadelphia, PA, USA; 42 Department of Neurology, Brigham and Women's Hospital, Boston, MA, USA; 43 Department of Genetics, Children’s Hospital of Eastern Ontario, Ottawa, ON, Canada

**Keywords:** RNU4ATAC, minor spliceosome, MOPD type 1, Roifman syndrome, Lowry-Wood syndrome

## Abstract

**Purpose:**

We aim to better define the genotype and phenotype spectrum of RNU4ATAC-opathy, demonstrate the utility of RNA sequencing for variant classification, and highlight challenges in detecting variants in this noncoding gene.

**Methods:**

Sixty individuals with molecularly confirmed RNU4ATAC-opathy were recruited from multiple clinical and research centers internationally. RNA sequencing was available for seven affected individuals.

**Results:**

We report the clinical and molecular findings of 60 individuals, including 42 not previously described, and 33 distinct *RNU4ATAC* variants, 13 of which are novel. Core features in this cohort—present in most individuals assessed and varying in severity—include microcephaly, short stature, skeletal anomalies, developmental delay, cerebral anomalies, skin conditions and immune deficiency. Additional findings such as diabetes, holoprosencephaly, and absence of various core features in some individuals highlight the broad phenotypic spectrum. All individuals with RNA sequencing showed a consistent pattern of minor intron retention. In six, RNA-seq enabled reclassification of variants of uncertain significance as likely pathogenic. While *RNU4ATAC* variants are generally covered by clinical exomes, they are often overlooked in analysis due to the noncoding nature.

**Conclusion:**

This study further highlights the variability of phenotypes and genotypes associated with RNU4ATAC-opathy. Laboratories should ensure *RNU4ATAC* and other noncoding genes are appropriately assessed by their analysis pipelines.

## Introduction

The U12-dependent minor spliceosome is an RNA:protein complex responsible for the removal of minor introns, which constitute approximately 0.5% of all introns in the human genome.^1,2^ Minor introns are found in around 700 human mRNA transcripts, most of which contain only a single minor intron alongside multiple major introns that are excised by the U2-dependent major spliceosome.^3,4^ Minor introns have distinct splicing sequence motifs from major introns, and the minor spliceosome is specialized to recognize them using a partially distinct set of proteins and small nuclear RNAs (snRNAs).^5,6^ The reason why two separate splicing systems have been evolutionarily maintained in most multicellular organisms remains unknown.

The critical role of non-coding RNA genes in human disease is increasingly recognized, and components of both the major and minor spliceosomes have been linked to Mendelian disorders. In 2011, pathogenic biallelic variants in *RNU4ATAC* (HGNC:34016), encoding the minor spliceosome snRNA U4atac, were the first variants identified in a non-coding RNA spliceosome gene to cause human disease.^7^ Initially associated with microcephalic osteodysplastic primordial dwarfism type 1 (MOPD1) (OMIM#210710),^7,8^ these variants were then linked to Roifman syndrome (OMIM#616651),^9^ and Lowry-Wood syndrome (OMIM#226960).^10^ First described clinically by Taybi and Linder in 1967, MOPD1 has been characterized by severe pre- and postnatal growth failure, microcephaly, brain malformations, developmental delay, spondyloepimetaphyseal dysplasia, and early death^7,8,11,12^ - with immunodeficiency recognized later, likely contributing to disease severity.^13^ Causative variants were suggested to cluster in the 5’ stem-loop of the snRNA.^14^ Roifman syndrome, first described in the late 1990s,^15^ has overlapping features, with more clinically prominent humoral immune deficiency, and generally milder growth restriction, microcephaly, spondyloepiphyseal dysplasia and developmental delay. Retinal dystrophy is also commonly observed. Roifman syndrome has been associated with variants in the snRNA stem II domain.^9,16^ Lowry Wood syndrome, initially described in 1975,^17^ was soon after found to be allelic to MOPD1 and Roifman syndrome, with microcephaly and multiple epiphyseal dysplasia, along with variable short stature, developmental delay, eye involvement and subclinical immunodeficiency.^10,18^

As the genetic basis of these conditions became clear, it was recognized that individuals often presented with combinations and severity of features that did not fit classic diagnostic categories. Initially, before the three conditions were known to be allelic, milder cases were labeled as "mild MOPD1,"^19–21^ making genotype-phenotype correlations challenging today. The broad phenotypic variability associated with *RNU4ATAC* dysfunction is now increasingly recognized, with reports of affected individuals first detected through broad genetic testing who lack features that would typically prompt clinical consideration of this condition.^22,23^ However, clinical recognition of this condition is also challenging because of this phenotypic heterogeneity. More recently, umbrella terms, *RNU4ATAC* spliceosomopathy or spliceopathy,^24,25^ RNU4ATAC-associated disorder,^22^ and RNU4ATAC-opathy^23,26,27^ have been suggested to better capture the full range of presentations linked to pathogenic variants in this gene, and support a more flexible, gene-centered diagnostic approach.

Most disease-associated variants in *RNU4ATAC* cluster in regions that interact with other components of the minor spliceosome, either non-coding RNAs (e.g., Stem II domain variants) or associated proteins (e.g., variants in the 5' Stem-Loop and Sm-protein binding domains).^14^ These variants lead to impaired assembly of the minor spliceosome and decreased excision of minor introns, but in most cases, only mild reductions in levels of correctly-spliced mRNA.^3,4^ However, RNA sequencing analysis on blood and other tissues from individuals with RNU4ATAC-opathy has revealed significant retention of minor introns.^9,25,28^ The mechanism by which these splicing defects result in the severe phenotypes seen in RNU4ATAC-opathy remains unknown. One hypothesis is that minor splicing may serve as a "molecular switch", regulating groups of functionally related genes at specific developmental stages.^3,29^ Testing this hypothesis will require the development of animal models of disease-associated variants in minor spliceosome components, as constitutive loss of these factors is embryonically lethal.

Several challenges complicate the detection and analysis of *RNU4ATAC* variants. First, *RNU4ATAC* is not always routinely included in clinical exome analysis.^23^ Inclusion of *RNU4ATAC* on exome capture kits is inconsistent; for example, only about 35% of exomes in gnomAD v4.1 cover *RNU4ATAC* at greater than 5X depth. However, this may not be representative of clinical exome sequencing, which typically provides better inclusion of disease-associated transcripts.^30^ Second, even when *RNU4ATAC* is adequately covered by exome or genome sequencing, variants may be filtered out during analysis because *RNU4ATAC* encodes a noncoding transcript. Standard analysis pipelines prioritize missense and predicted loss-of-function variants, often overlooking noncoding variants —particularly those in genes encoding a functional RNA like *RNU4ATAC*. Lastly, variant nomenclature presents another challenge. The genomic coordinates for its first nucleotide differ by one base pair between Ensembl (chr2:121,530,881–121,531,007 [hg38], ENST00000580972.1) and NCBI (chr2:121,530,880–121,531,009 [hg38], NR_023343.3). The MANE Select transcript for *RNU4ATAC* was only very recently introduced and is not yet implemented into many databases. This discrepancy can lead to confusion during variant interpretation and contribute to errors in variant annotation and reporting.

In this study, we aim to better characterize the genotype and phenotype spectrum of RNU4ATAC-opathy, demonstrate the utility of RNA sequencing in variant classification, and highlight the specific challenges associated with detecting and analyzing variants in this gene. We describe 60 affected individuals, including 42 not previously reported, and 33 distinct variants, 13 of which have not been previously reported in the literature.

## Methods

### Case selection

Individuals with molecularly confirmed RNU4ATAC-opathy were recruited from multiple clinical and research centers. This collaborative effort was initiated following a meeting of clinicians with a shared interest in spliceosomopathies at the David W. Smith Workshop on Malformations and Morphogenesis in August 2023. The affected individuals were participants in various rare disease research programs, including the NHGRI Genomics Research to Elucidate the Genetics of Rare diseases (GREGoR)^31^ consortium at the Broad Center for Mendelian Genomics and Stanford sites, the Undiagnosed Diseases Network (UDN),^32^ and the King Faisal Specialist Hospital and Research Centre.^33^ Additional individuals were enrolled through the Primordial Dwarfism Registry, at Nemours Children’s Hospital, Delaware. Other individuals were enrolled through the Microcephaly Primordial Dwarfism research study at the MRC Human Genetics Unit, University of Edinburgh, UK, approved by the Multicentre Research Ethics Committee for Scotland (05/MRE00/74), and by the Leeds (East) Research Ethics Committee (reference no. 10/H1307/2; Integrated Research Approval System project ID: 62971). Some individuals were also included in the study following the identification of *RNU4ATAC* variants through clinical genetic testing and subsequent communication between clinical teams. Clinical information was uniformly obtained from each clinical or research team using a structured phenotypic table (Summarized in supplemental Table 1).

Ethical approval for the study was obtained from all participating institutions, including the Stanford University IRB (protocol #60837), MassGeneralBrigham (protocol 2016P001422), Boston Children’s Hospital (10-02-0053), Nemours Children’s Hospital (protocol #83142), University of North Carolina at Chapel Hill (IRB #21-1240), the King Faisal Specialist Hospital and Research Centre (protocol 2210029 and 2080006), the Multi-centre Research Ethics Committee for Scotland (05/MRE00/74), the Leeds (East) Research Ethics Committee (10/H1307/2; IRAS 62971), and Nationwide Children’s Hospital (STUDY00000312). Informed consent was obtained from all participants or their legal guardians. Consent for publication of clinical data and photographs was obtained under approved protocols at the National Human Genome Research Institute, NIH (protocol 15-HG-0130), Stanford University (protocol #60837), Boston Children’s Hospital (10-02-0053), MassGeneralBrigham (protocol 2016P001422) and Nemours (protocol #83142).

### DNA Sequencing

Several individuals, especially those enrolled in the Primordial Dwarfism Registry and the Microcephaly Primordial Dwarfism research study, presented with clinical features suggestive of RNU4ATAC-opathy and were diagnosed after single gene testing by Sanger sequencing (primers available on request). Additional individuals underwent exome or genome sequencing as part of research studies focused on undiagnosed rare genetic conditions.^31–33^ In other cases, *RNU4ATAC* variants were identified through clinical genetic testing performed by commercial laboratories, including gene panels and clinical exome sequencing. Specific sequencing methodologies employed for each individual are detailed in Supplemental Table 2.

### RNA Sequencing

RNA-seq was performed on samples from seven individuals at the Broad CMG and Stanford sites of the NHGRI GREGoR consortium. Whole blood RNA-seq was conducted for six individuals (ID002, ID011, ID027, ID056, ID057 and ID058), and one individual (ID001) underwent RNA-seq on a muscle biopsy sample. Aberrant splicing events were analyzed using the outlier detection methods FRASER^34^ and FRASER2.^35^ FRASER and FRASER2 analysis for individuals ID001 and ID002 were performed at the Broad NHGRI GREGoR Consortium site, using tissue-matched heterogenous rare disease cohorts comprising 190 muscle samples and 104 blood samples, respectively. Whole blood RNA-seq for individuals ID011, ID027, ID056, ID057, and ID058 was performed at the Stanford NHGRI GREGoR consortium site, using whole blood samples from a rare disease cohort of 462 individuals. The experimental protocol for all 462 whole blood samples was previously reported in Arriaga *et al*., 2025, including analysis and methodological definition of excess intron retention in minor intron-containing genes.^25^ Additional methodological details are available in Arriaga *et al*.^25^

### Variant classification

NCBI transcript was used (NR_023343.1) (NC_000002.12: Chr2:121,530,880–121,531,009 [GRCh38]). Variants were classified according to the ACMG/AMP criteria^36^ and also specifically following the recommendations from Ellingford *et al*^37^ for variants found in non-coding regions of the genome (Supplemental Table 2). We applied PM2_Supporting to variants that were absent or very rare in gnomAD v4.1.0 (highest minor allele frequency <0.01% and no homozygous individuals). PS3 (for well-established functional assay) was applied when RNA-sequencing analyses identified significant minor intron retention events, or when other functional assays provided evidence supporting pathogenicity. PM1 was applied to variants located within known mutational hotspots of *RNU4ATAC*, specifically the Stem II region (NR_023343.1:3-18), the 5’ stem-loop (28-55), and the Sm protein-binding site (111-124). We applied PM3 to evaluate the individual’s variant and previously reported cases with the same variant, PP1 to assess segregation of the variant with the condition within families, and PS1_moderate when another variant with a different nucleotide change at the same variant location was classified as pathogenic.

## Results

The study consisted of 60 individuals, 29 females and 31 males, from 54 families. The individuals ranged in age from prenatal to 24 years of age. The mean age was 8.2 years and median age was 6.0 years at last evaluation. Ancestry and self-reported ethnicity were not collected. Clinicians classified individuals into a phenotypic subcategory based on their own judgment, and on the known *RNU4ATAC* clinical diagnoses at the time - which was only MOPD1 until 2015. The most common was MOPD1 (36/60, 60%) followed by RNU4atac-opathy (18/60, 30%), Roifman syndrome (5/60, 8.3%), and Lowry Wood syndrome (1/60, 1.7%).

### Characteristics of affected individuals

#### Craniofacial dysmorphology

**Figure 1:**
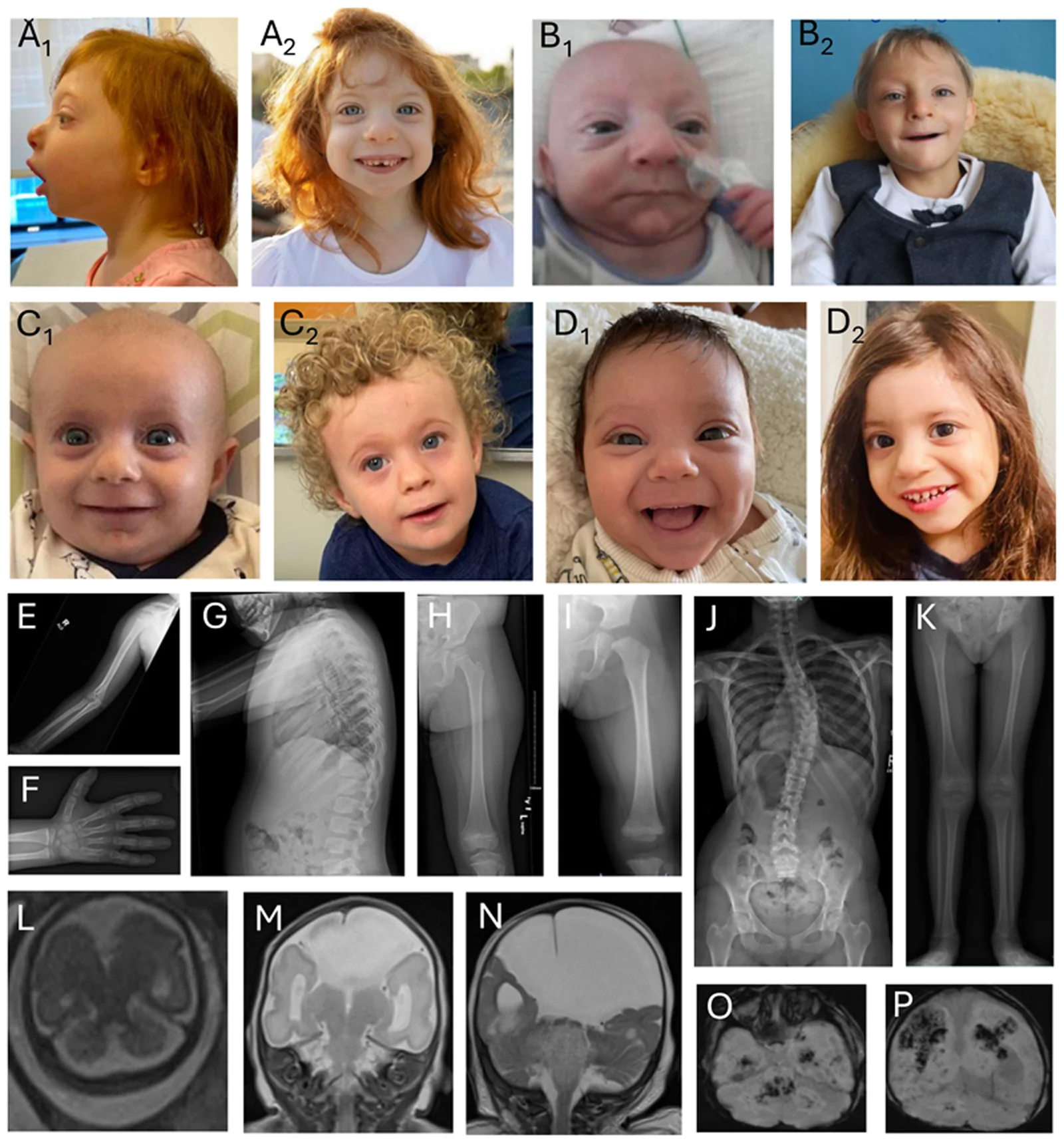
Photographs and imaging of individuals with RNU4ATAC-opathy. **A-D**. Facial features of four individuals with RNU4atac-opathy. Note the microcephaly, prominent eyes with epicanthus, infraorbital creases, slightly arched eyebrows, pointed chin, bulbous nasal tip, low set ears, and wide smile with slightly widely spaced teeth and thin upper vermilion of the lip. **A**_**1**_**/A**_**2**_. ID002 at 2 years and 3.5 years of age. Also note the mild retrognathia. **B**_**1**_**/B**_**2**_. ID016 at 3 months and 5.5 years of age. **C**_**1**_**/C**_**2**_. ID026 at 4 months and 2 years of age **D**_**1**_**/D**_**2**_. ID052 at 2 months and 4 years of age **E-H**. Radiographs of a 6-year-old female (ID001). Despite skeletal involvement in most individuals, no skeletal dysplasia was identified in the upper extremities (E), hands (F), spine (G), or lower extremities (H). **I**. AP lower extremity radiograph of a 2-year-old female (ID002), showing epiphyseal dysplasia identified after confirmation of the RNU4ATAC-opathy diagnosis. **J**. AP spine radiograph of a 17-year-old female (ID019), demonstrating thoracic scoliosis. **K**. AP lower extremity radiograph of a 6-year-old female (ID023), showing subtle mesomelia, epiphyseal dysplasia, and metaphyseal involvement **L-P**. MRI images of a male (ID013) at various ages. **L**. Fetal MRI at 28 weeks’ gestation showing lobar holoprosencephaly with significantly underdeveloped frontal, parietal, and occipital lobes **M/N**. Coronal T2 images at 2 weeks of age (N) showing a large dorsal intracranial cyst splaying the cerebral hemispheres, and at 12 months (O) showing cyst progression. **O**. Axial SWI at 12 months showing microhemorrhages in the brainstem, temporal lobes, and cerebellum. **P**. Coronal SWI at 12 months showing microhemorrhages in the temporal lobes, basal ganglia, and cerebellum.

Of the 39 individuals with available craniofacial morphology data, all but one was noted with dysmorphic features (Figure 1). These features were variable, but commonly included forehead differences (11/39, 28%), such as sloping or prominent forehead; prominent eyes (7/39, 18%), proptosis, hypo or hypertelorism, and downslanting palpebral fissures; and nasal differences, with a bulbous tip reported in eight individuals and nasal bridge variations in 11 (28%). Micrognathia was observed in seven individuals, and minor ear anomalies were present in 49% (19/39), including small ears in five, and features such as thin or simplified helices, large ears, low-set and posteriorly rotated ears, and small earlobes.

#### Growth

**Figure 2:**
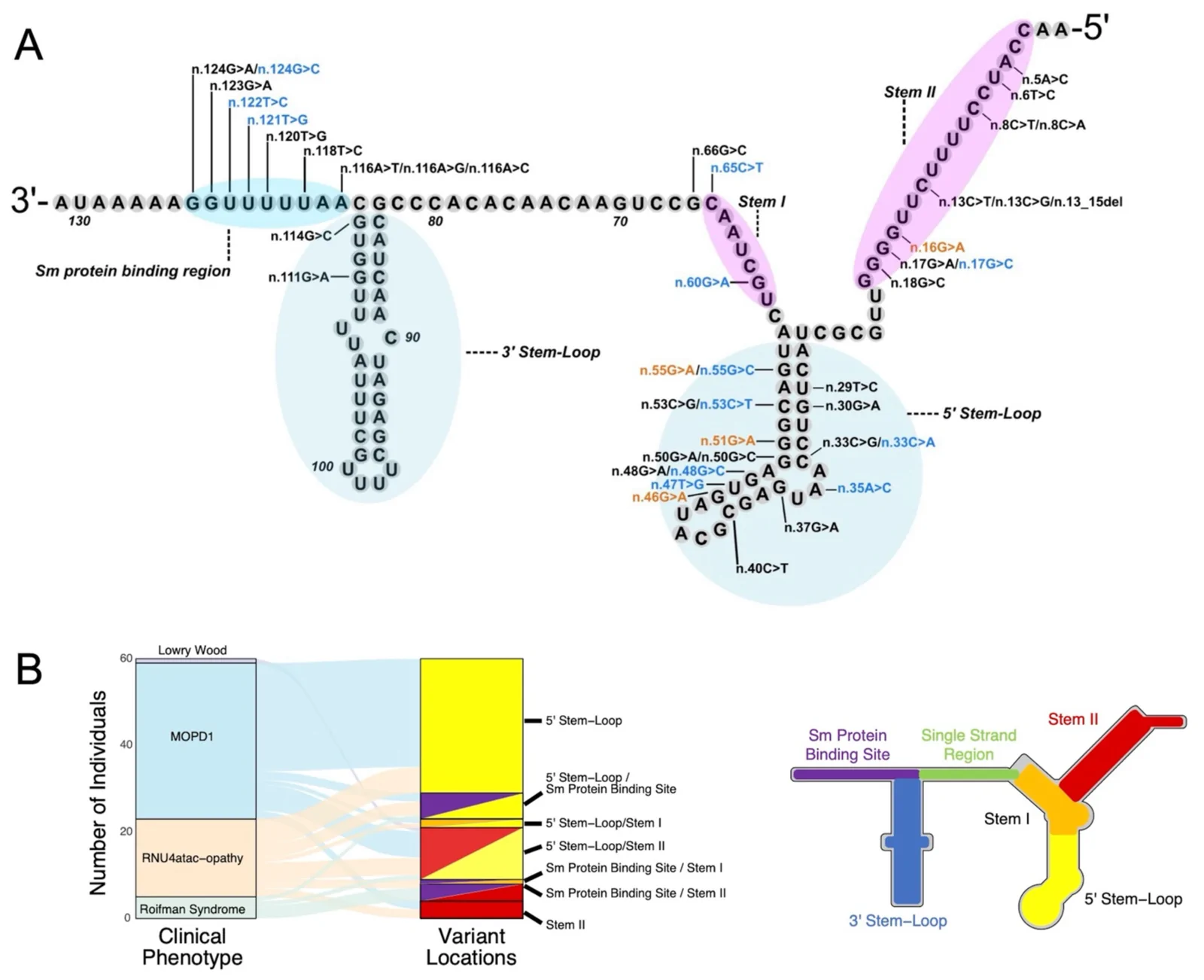
Growth and clinical features of individuals with RNU4ATAC-opathy in this cohort. **A**. Phenotype square plot with each box denoting one individual. The boxes with color signify affected, dark gray signifies not affected, and light gray signifies data not available (NA). IUGR: Intrauterine growth retardation; GI: gastrointestinal; GU: genitourinary. **B**. Growth parameters at age of last evaluation in standard deviation (SD) for weight, height, and head circumference. The phenotypic subcategories were assigned by clinicians, and microcephalic osteodysplastic primordial dwarfism type I (MOPD1) is shown in comparison to all subcategories (Lowry Wood syndrome, RNU4atac-opathy, and Roifman syndrome).

Growth was affected in most individuals (Figure 2A). Intrauterine growth restriction (IUGR) was noted in 75% (36/48). Microcephaly and short stature were reported in almost all individuals at 95% (56/59, 55/58 respectively). Of note, one individual had both average stature and average head circumference. At the time of last evaluation, 92% (45/49) of individuals had microcephaly (head circumference ≥2 SD below the mean), with nearly half (23/49, 47%) measuring -5 to -10 SD below the mean. Short stature was common, observed in 86.8% (46/53) of individuals at their last evaluation. Of these, 5 had heights between -2 and -2.5 SD, 16 between -2.5 and -5 SD, 19 between -5 and -10 SD, and 6 had extreme short stature below -10 SD. Low weight (≥2 SD below the mean) was also present in 80% (36/45) of individuals, though less severely affected compared to height, with most individuals in the -2.5 to -5 SD range (18/45, 40%). Notably, no individual had weight below -10 SD, while 11.3% (6/53) had length in that range and 20.4% (10/49) had head circumference below -10 SD. All individuals with a height below -7.5 SD had MOPD1 (Figure 2B). Overall, individuals with MOPD1 were shorter, weighed less, and had smaller head circumferences than those in other phenotypic subgroups (Figure 2B).

#### Neurodevelopment and neurological anomalies

Developmental delay was present in the majority of individuals, with 92.5% (37/40) reported to have some degree of delay (Figure 2A). The degree of delay was documented in 35 individuals: 10 had mild delay, 11 had moderate, two had severe, and nine had profound. Notably, 8.6% (3/35) were reported to have no history of developmental delay but were diagnosed with mild intellectual disability (ID) in adolescence. Information on intellectual functioning was available for 26 individuals, with 96.2% (25/26) reported to have ID. The level of functioning was specified in 20 individuals, including one who was specifically noted not to have ID. Among the remaining individuals, 13 had mild ID, two had moderate, two had severe, and two had profound.

Neurologic manifestations were common, with 91.5% (43/47) of individuals exhibiting a cerebral anomaly and 33.3% (13/39) reported to have seizures (Figure 2A). Among those with seizures and available imaging (12/13), all were found to have a cerebral malformation on brain MRI. Common anomalies included corpus callosum aplasia/hypoplasia in 25, simplified gyral pattern in 10, Dandy-Walker variant in three, holoprosencephaly in three, and lissencephaly in nine. Intracranial cysts were present in eight, with arachnoid and interhemispheric locations being the most frequent, one of which caused hydrocephalus requiring treatment by ventriculoperitoneal shunt. Notably, stroke was observed in three, all in a nonvascular distribution and each associated with significant brain malformation and disorganization. The three individuals who had a stroke were either homozygous or compound heterozygous for variants exclusively involving n.50G>A, n.50G>C, and n.51G>A.

#### Skeletal anomalies

Skeletal involvement was a key but not universal feature, with 87.8% (43/49) of individuals showing anomalies on physical examination (Figure 2A). Among those with documented assessments of body proportions, 44.4% (12/27) were described as proportionate. Of those with disproportion, 80% (12/15) had mesomelia, while two individuals had micromelia and one had acromelia; none had rhizomelia. A variety of skeletal findings were reported, many of which were mild, including brachydactyly in 11, tapered fingers in eight, and clinodactyly in seven. Additional findings included pes planus, and joint abnormalities, such as hypermobility, dislocations, and contractures.

Approximately half of the cohort underwent skeletal imaging, though not all were comprehensive. Among those assessed, skeletal anomalies were identified in 88% (30/34) (Figure 1). The spine (20/31) and epiphyses (19/28) were commonly affected, and to a lesser extent, the metaphyses (13/28). Combinations of skeletal dysplasia were noted beyond that classically described, including spondyloepimetaphyseal dysplasia in those not clinically diagnosed with MOPD1 and spondyloepiphyseal dysplasia beyond those clinically diagnosed with Roifman syndrome. Additionally, scoliosis was noted in six, atlantoaxial instability was identified in one, and hip dysplasia with hip replacement in young adulthood was noted for an additional individual. Of the three individuals in the cohort with average stature, two had no skeletal dysplasia and one was not assessed for this. Notably, one individual with no skeletal anomalies on clinical examination was found to have skeletal dysplasia on screening radiographs.

#### Immune function

Immune deficiency was described in a majority of patients for whom data was available (27/35, 77%) (Figure 2A). The majority were noted to have hypogammaglobulinemia (17/27, 63%), three with lymphopenia and one individual with common variable immunodeficiency (CVID). Most (17/27, 63%) were treated with immunoglobulin replacement therapy (IVIG). Recurrent infections were reported in seven, with recurrent pulmonary infections noted in four, though this may underestimate the true prevalence due to how data were collected as the focus was on immune deficiency rather than infection history. A sibling pair (one male, one female) each experienced chronic inflammatory demyelinating polyneuropathy (CIDP), one treated with IVIG and Rituximab and one treated with IVIG.

#### Dermatologic findings

Skin conditions were observed in 76.2% (32/42) (Figure 2A), with eczema being the most common feature in 21, followed by dry skin in 14. Ichthyosis and ectodermal dysplasia were each reported in two individuals. Hair anomalies were less common, with alopecia and sparse hair reported in two individuals each, and hirsutism in three. Peripheral edema was also reported in four individuals, most often affecting the feet, although one individual had involvement of the hands.

#### Other medical issues

Ophthalmologic anomalies were common in this cohort. Retinal abnormalities were observed in 41% of individuals (16/39) (Figure 2A), including pigmentary degeneration or retinitis pigmentosa in five individuals, salt-and-pepper fundus in two, vascular tortuosity and peripapillary chorioretinal atrophic conus each in one individual. Strabismus was identified in 59% (23/39). Additional findings included myopia in nine individuals, cataract and cortical visual impairment in four individuals each.

Hearing loss was present in 36% (14/39) of individuals with all types identified (Figure 2A). Of the 14 individuals with hearing loss, six had sensorineural hearing loss, two had conductive hearing loss, and six had unspecified hearing loss.

Endocrine data were available for 50% of the cohort (30/60), of whom 50% (15/30) had reported endocrine anomalies (Figure 2A). Among those with endocrinopathy, the most common issues were hypothyroidism in eight, diabetes mellitus in six—with type 1 in all but one case—growth hormone deficiency in two, and adrenal insufficiency in three.

Minor cardiac malformations were observed in 22.9% (8/35) of the cohort (Figure 2A), including atrial septal defects, ventricular septal defects, patent foramen ovale, or patent ductus arteriosus. All cases showed resolution or improvement on follow-up imaging without the need for surgical intervention. An additional individual was diagnosed with heart failure at 18 years of age and was found to have a pathogenic MT-TL1 variant, which may have been causative.

Renal anomalies were identified in 37.1% (13/35), although few structural anomalies were observed (Figure 2A). The most common findings on renal ultrasound were echogenic kidneys in three and renal cyst(s) in two. Hypertension was present in three, including one individual with renal artery stenosis. Electrolyte abnormalities were also reported in three, with renal tubular dysfunction specifically noted in one individual. Notably, two developed hemolytic uremic syndrome (HUS), and two individuals were diagnosed with chronic kidney disease (CKD).

Genitourinary anomalies were identified in 31.4% (11/35) of the cohort (Figure 2A), all of whom were males. Among these, six had cryptorchidism, five had micropenis, and one had phimosis. One individual with micropenis also had ambiguous genitalia and retractile testes.

Gastrointestinal anomalies were reported in 60.6% (20/33) (Figure 2A), with dysphagia being the most common in seven. Other findings included reflux in six, constipation in five, inguinal hernia in three, feeding difficulties in five, and single cases of lymphocytic esophagitis and eosinophilic esophagitis. Notably, four individuals required gastrostomy tubes, with at least two being G-tube-dependent and receiving minimal or no oral nutrition.

#### Mortality

Twelve individuals in the cohort are known to be deceased (12/60, 20%), including one pregnancy termination. Age at death ranged from 32 weeks gestation to 12 yrs. The most commonly identified causes of death were sepsis and respiratory failure (3 cases each). No further details were available for the remaining (aside from pregnancy termination). Two individuals had holoprosencephaly. One infant with semilobar holoprosencephaly and vermian hypoplasia died on day one of life due to respiratory failure. Another individual with lobar holoprosencephaly and complex MRI anomalies (Figure 1) died at 12 months from respiratory and multi-organ failure following a stroke. All individuals in this cohort with reduced lifespans carried either homozygous or compound heterozygous variants exclusively involving n.50G>A, n.50G>C, n.51G>A, or n.55G>A.

### Genotype spectrum in the cohort

**Figure 3:**
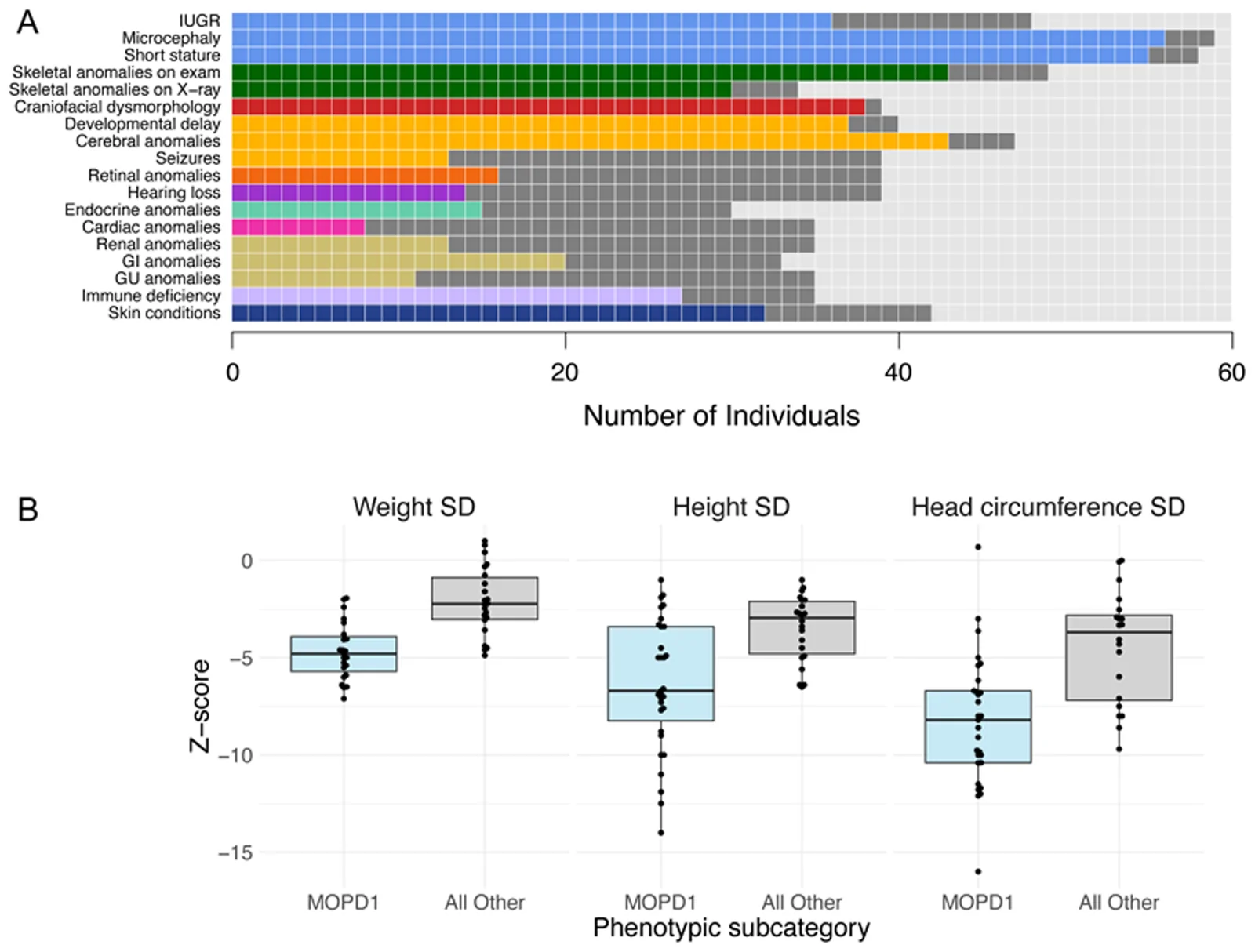
U4ATAC snRNA molecule with annotated variants and alluvial plot showing variant distribution. **A**. The U4atac molecule is represented with reported variants in affected individuals from the literature and in this cohort. Variants present in this cohort that have not been previously reported in the literature are shown in blue. Variants that have been reported in the homozygous state are shown in orange. The 85-nt duplication (n.16_100dup) described by Krøigård et al. (2016) (also Individual ID032 and ID033 in this cohort) is not shown. Adapted from Benoit-Pilven et al. (2020). NCBI transcript was used (NR_023343.1) (Chr2:121,530,880–121,531,009 [hg38]) **B**. The left column indicates the number of individuals and their clinical phenotype/subcategory. The right column shows the variant locations, color-coded to match the regions on the U4atac diagram on the right. These data demonstrate that phenotypic subcategories in this cohort are associated with variants across multiple locations and that variants typically linked to one clinical phenotype are also found in other subcategories.

A total of 33 distinct variants were identified in this cohort, 13 of which have not been previously reported in the literature (Figure 3A). The most frequent variant in the cohort was n.51G>A, found in 16 individuals, followed by n.55G>A in 12 individuals, n.13C>T in 10, n.40C>T in 9, and n.46G>A in 7. Across all variants, the 5′ Stem Loop was the most common variant location.

Nine variants were classified as pathogenic, 18 as likely pathogenic, and 6 as high-interest VUS. All individuals carrying a VUS were compound heterozygotes for a pathogenic or likely pathogenic variant. All these VUS were considered highly likely to be causative for the individual’s phenotype: this judgement is based on the combination of clinical phenotypes consistent with RNU4ATAC-opathy and variants that are expected to disrupt spliceosome RNA function through altered folding, loss of interactions with RNA/protein partners, or both. These functional considerations specific to small, structured RNAs are not well considered in current variant classification guidelines. Individuals with these VUS were therefore considered diagnosed despite more evidence being needed at the variant level to fulfill current criteria for pathogenicity.

### Genotype-phenotype analysis

In this cohort, 8 individuals were homozygous for the n.51G>A variant, all classified as having MOPD1. Looking at the phenotypes across this subcohort, neurodevelopmental data were available for two: one had profound impairment, and the other was noted to have developmental delay without further detail. Brain MRI data were available for seven of the eight individuals, all showing cerebral malformations: two with isolated agenesis of the corpus callosum (ACC), one with an interhemispheric cyst, one with a thin immature cortex, and three with complex findings (e.g., semilobar holoprosencephaly; simplified gyral pattern with ventriculomegaly, heterotopia, partial ACC; and lissencephaly with atrophy and ACC). X-rays were available for five individuals, all with skeletal abnormalities. One individual had hypogammaglobulinemia; immune data were unavailable for the others. Of the five with mortality data, four were deceased. Growth was severely affected, with mean height and head circumference of -8.62 SD and -10.2 SD, respectively, compared to -5.2 SD and -6.8 SD in the overall cohort (Supplemental Figure 1). Heterozygous variants around the 5’ Stem Loop were also associated with more severe short stature and microcephaly, suggesting a genotype-phenotype correlation for growth (Supplemental Figure 1).

The recurrent n.51G>A variant was also observed in a compound heterozygous state with other variants and across different phenotypic subcategories, including RNU4ATAC-opathy and Lowry Wood syndrome. Many of the individuals categorized as MOPD1 carried two variants in the 5’ Stem-Loop. However, variants associated with MOPD1 were also seen in a variety of other locations outside the 5’ Stem-Loop (Figure 3B). The n.13C>T variant from the Stem II region was identified in individuals across multiple phenotypic subcategories as well, most commonly in Roifman syndrome, but also in several cases of MOPD1. Notably, none of the individuals with Roifman syndrome in our cohort had variants located exclusively in Stem II, which has been considered a critical region for Roifman syndrome. In fact, one individual had no variants in Stem II, but rather in the Sm Protein Binding Site and the 5′ Stem-Loop (e.g., n.124G>C/n.40C>T) (Figure 3B). These findings underscore the challenge of drawing clear genotype-phenotype correlations in this autosomal recessive condition due to the broad range of variant locations linked to each phenotypic subcategory. Also, as in many recessive disorders, the less severe allele may determine the clinical presentation, likely modulating the phenotypes observed here.

### RNA sequencing

**Figure 4:**
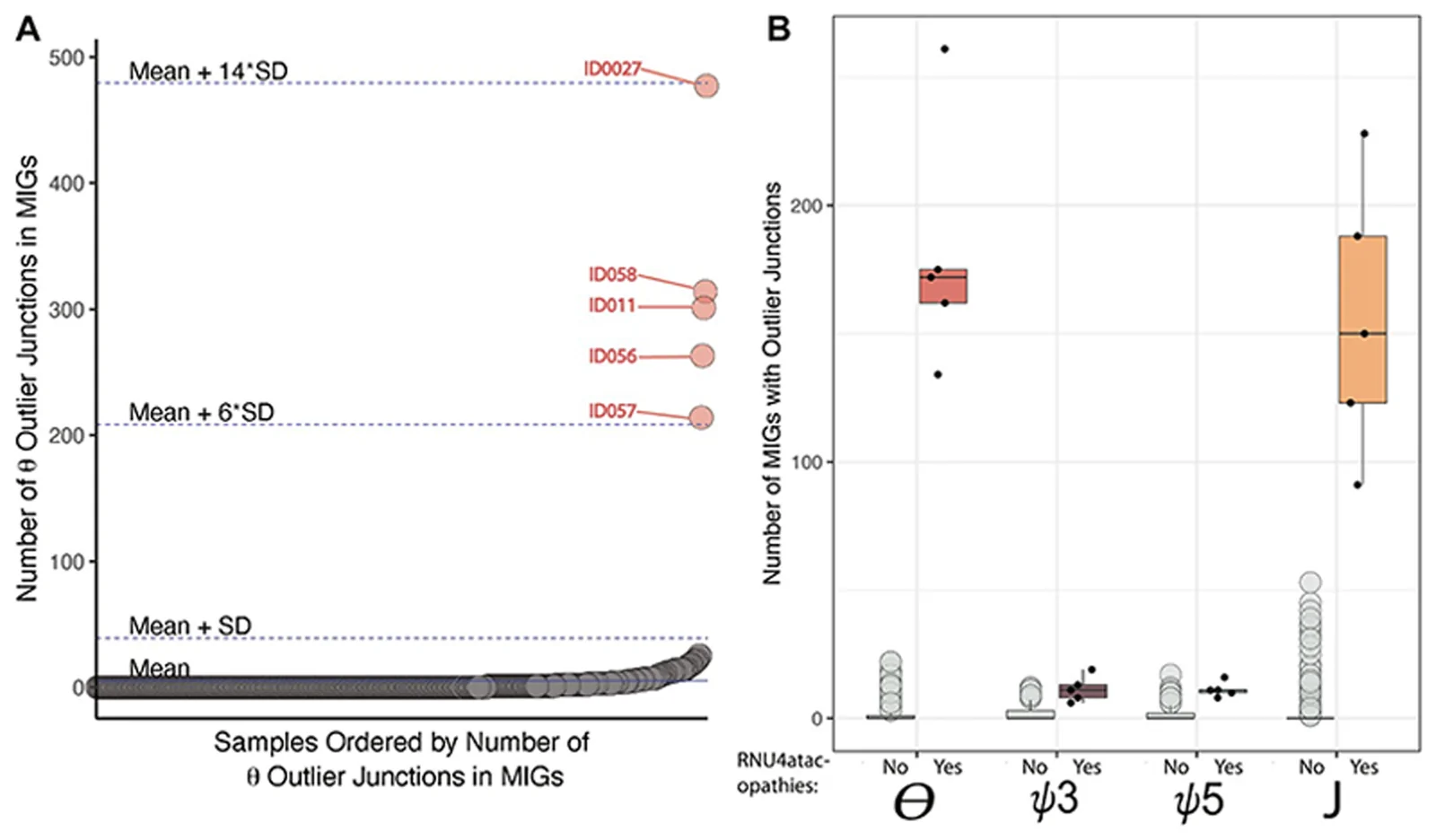
Whole blood RNA sequencing results of five individuals with RNU4ATAC-opathy showing excess intron retention outliers in minor intron containing genes (MIGs) compared to a rare disease blood RNA sequencing cohort. **A**. Plot showing the number of significant (q < 0.05, abs(Δ**Ψ**) > 0.3) intron retention (*θ*) events in minor intron containing genes (MIGs). Each dot represents an individual from our whole blood cohort, and the Y-axis position represents the number of significant intron retention events in MIGs detected in that individual. The X-axis is ordered by the number of significant intron retention events in MIGs. Five individuals with RNU4ATAC-opathy from this cohort (red dots, labeled by identifier) were outliers, each showing a number of intron retention events in MIGs greater than two standard deviations above the mean. **B**. This boxplot, read from left to right, shows the number of MIGs with significant splicing outliers for each outlier type: θ (intron retention), ψ3, ψ5, and Jaccard index (J). For each type, the gray boxplot labeled "No" on the left represents individuals without rare variants in the minor spliceosome. The colored boxplot labeled "Yes" on the right (red for θ, purple for ψ3, blue for ψ5, and orange for J) corresponds to individuals with RNU4ATAC-opathy. The black circles on these colored boxplots represent the number of MIGs of the specified outlier type found in the individuals with RNU4atac-opathy.

The seven individuals in this cohort for whom we have RNA sequencing data showed a significant excess of intron retention events, represented as *theta* outliers by FRASER, specifically in minor intron containing genes (MIGs) (Figure 4A).^34^ These samples showed greater than 40-fold significant intron retention events in MIGs compared to the overall mean of their tissue-matched rare disease cohort. Specifically, the five individuals sequenced at the Stanford GREGoR site (ID011, ID027, ID056, ID057, and ID058) contained a mean of 273 and median of 282 (IQR[251-304]) intron retention events in MIGs, while the rest of the cohort had a mean of 3.51 and a median of 0 (IQR[0-1]) (Figure 4B).

Additionally, the two individuals sequenced at the Broad GREGoR site (ID001 and ID002) showed an excess of intron retention in MIGs (one each from the muscle and whole blood RNA-seq cohorts, respectively) (Supplemental Figure 2). The ID001 sample had 171 significant intron retention events in MIGs, compared to a median of 4 (IQR[2-9]) in the cohort of 190 muscle samples. The ID002 sample had 291 significant intron retention events in MIGs, compared to a median of 5 (IQR[2-8]) in the cohort of 104 whole blood samples. As was seen in the Stanford site, the individuals sequenced at the Broad site showed greater than 40-fold significant intron retention events in MIGs, and approximately 60-fold increase in the whole blood samples across both sites.

There was no significant difference in the RNA quality (RIN) between these seven individuals compared to the rest of the cohort. Other splicing metrics from the FRASER method, such as psi3 and psi5, which represent alternative donor and acceptor usage, showed no significant difference of outliers in MIGs between the affected individuals and the remainder of the rare disease cohort (Figure 4B). This highlights the specificity of splicing outlier pattern to minor intron retention events in MIGs.

Of the seven individuals who underwent RNA sequencing, six initially had a likely pathogenic variant in trans with a VUS, and one had two likely pathogenic variants in trans. RNA sequencing enabled reclassification of the six VUS as likely pathogenic, confirming a molecular diagnosis in these individuals.

## Discussion

We present a large cohort of 60 individuals with RNU4ATAC-opathy, highlighting novel variants, RNA sequencing findings, phenotypic variability, underreported features, and management considerations.

Previously reported findings were common in this cohort including pre- and postnatal growth deficiency and skeletal anomalies. Nearly all individuals had at least subtle skeletal anomalies on physical examination, mostly of the hands and feet, or showed disproportionate short stature. Even when physical findings were minimal, most individuals who underwent radiography showed evidence of skeletal dysplasia. Interestingly, four individuals had normal X rays, all of whom were of average stature or borderline short stature, demonstrating that skeletal anomalies are not always present. Nonetheless, our findings support spine and lower extremity radiographs in individuals with confirmed or suspected RNU4atac-opathy, particularly for those with short stature, noting that radiographic abnormalities may emerge with age, and skeletal dysplasia could be present without signs on physical examination.

Developmental delay was common in this cohort and showed marked variability, ranging from mild to profound. Neurologic anomalies were frequent, including brain malformations of various severities —most often involving the corpus callosum. More complex malformations and disorganization, including lissencephaly and holoprosencephaly, were seen exclusively in individuals classified as MOPD1, as were strokes that did not follow a vascular pattern. As expected, profound developmental delay was also correlated with more significant brain anomalies. Interestingly, the degree of microcephaly was not predictive of cognition level. Over one third of individuals (13/35) experienced seizures, almost all of whom had significant anomalies on brain imaging. Brain MRI and referral to neurology should be considered when clinically appropriate for all individuals with confirmed or suspected RNU4atac-opathy.

Immunodeficiency, and more specifically hypogammaglobulinemia, was observed in many individuals in this cohort for whom data was available and was often only identified after genetic diagnosis and targeted immunological assessment. Importantly, this immune phenotype extended beyond individuals clinically diagnosed with Roifman syndrome. Immune evaluation should be performed in all individuals with confirmed or suspected RNU4atac-opathy.^23^ As immune deficiency requiring treatment may emerge over time, ongoing monitoring is recommended.

Endocrinopathies were also frequent and often required treatment, including hypothyroidism, diabetes (most commonly Type 1), growth hormone deficiency, and adrenal insufficiency. Clinicians should maintain a low threshold for investigating endocrine abnormalities and initiate appropriate workup when indicated. It is interesting to highlight that several individuals had auto-immune endocrinopathies. Recent work has reported dysregulated type 1 interferon signaling in individuals with RNU4ATAC-opathy.^38^ Together, these findings suggest that *RNU4ATAC* variants may lead to broader immune dysregulation, expanding beyond impaired B cell development.

In addition, involvement of other organ systems was common, reinforcing recent recommendations for initial evaluations in individuals with confirmed or suspected RNU4ATAC-opathy.^23^ The following should be considered: an echocardiogram, genital examination (particularly in males), baseline renal function test (e.g., BUN, creatinine), and renal ultrasound. In addition, baseline ophthalmologic and audiologic evaluations are recommended with periodic subsequent examinations as both retinal pathology and hearing loss may develop over time. Ongoing monitoring for feeding issues, growth, and development is also recommended.

Overall, the core features—present in most individuals with RNU4ATAC-opathy in this cohort and varying in severity—include microcephaly, short stature, skeletal dysplasia, developmental delay, variable dysmorphisms, cerebral anomalies, skin conditions and immune deficiency. Retinopathies, hearing loss, and endocrinopathies may be underreported in this cohort, as these features can emerge later despite normal initial screenings. These data highlight that RNU4ATAC-opathy does not uniformly present with all core features; nonetheless, comprehensive baseline evaluations and longitudinal monitoring of affected systems are recommended.

This study has several limitations, mainly that clinical data were collected by multiple teams, resulting in variability in level of detail. Percentages were calculated only for individuals in whom the feature was assessed, which varied across the 60 cases due to differences in clinical evaluation and data collection. This may lead to under- or overestimation. Body proportion was difficult to assess consistently, especially when abnormalities may be subtle, and no standardized method was used. Some clinicians and radiologists had expertise in skeletal disorders, while others did not, so subtle findings could be missed for some. Many were also too young to assess later-emerging findings such as epiphyseal dysplasia, scoliosis, retinal dystrophy, hearing loss, endocrine anomalies, or intellectual disability.

In previous studies, MOPD1-associated variants were reported to cluster in the 5’ stem loop, also with compound heterozygous variants in Stem I, the Sm protein binding site, or the 3’ stem loop.^16^ Variants in Stem II, by contrast, were not linked to MOPD1 but were instead associated with Roifman syndrome, leading to Stem II being considered the “Roifman critical region”.^16^ However, in this cohort, we observed that variants in Stem II, when paired with a variant in the 5’ stem loop, were also associated with MOPD1 (Figure 3B). Importantly, interpretation is complicated by the lack of clear diagnostic criteria for providers to differentiate between these overlapping allelic conditions, and by phenotype subcategorization being performed by multiple clinical teams in this cohort. Notably, at least 22 individuals in this cohort were clinically diagnosed with MOPD1 when that was the only known clinical diagnosis associated with *RNU4ATAC* variants. At the time, individuals with milder features were often described as “mild MOPD1,” consistent with other publications of that time.^19–21^ Overall, our findings underscore the difficulty of establishing genotype–phenotype correlations in the context of ultra-rare, medically complex RNU4ATAC-opathy. That said, our results support that the n.51G>A variant—specifically in the homozygous state—is associated with a more severe phenotype.

This study highlights that while phenotypic variability exists across RNU4ATAC-related subcategories, significant overlap supports the view of a shared disease spectrum. All three conditions follow an autosomal recessive mode of inheritance, and differences in molecular mechanisms have not been established. In this cohort, variant location did not reliably distinguish subtypes, except for some homozygous variants in the 5’ stem loop associated with MOPD1. Given the difficulty in defining clear criteria to differentiate each phenotypic subcategory, we suggest adopting a gene-centered approach, such as “RNU4ATAC-opathy”, which provides greater flexibility and better reflects the wide range of severity and multisystem involvement observed clinically.

Importantly, the diagnosis of RNU4ATAC-opathy was missed on both research and clinical exomes in several individuals in this cohort and was only later identified through genome sequencing—or, in some cases, by targeted gene sequencing or panel. Given its broad phenotypic spectrum, *RNU4ATAC* should be included in all clinical exome pipelines, not just skeletal dysplasia or immune deficiency panels. Many features, such as IUGR or cerebral malformations, can present prenatally; therefore, *RNU4ATAC* should also be assessed appropriately in prenatal exome and panel analyses.

As *RNU4ATAC* is a non-coding gene, computational tools are currently poor predictors of variant impact.^14^ Current variant classification guidelines and computational tools are not designed to assess pathogenicity in small structured, functional RNAs like RNU4ATAC, making classification of potentially disruptive variants difficult. Further guidelines, bioinformatic tools and experimental approaches specifically tailored to these RNAs are needed. The broad phenotypic variability of RNU4ATAC-opathy further complicates this situation, underscoring the need for functional validation of VUS. As demonstrated in this study and by Arriaga *et al*., detection of transcriptome-wide intron retention events in MIGs, a signature unique to minor spliceopathies, can provide such validation. In this cohort, RNA-seq enabled variant reclassification in all seven individuals tested and confirmed a molecular diagnosis in six; the remaining individual had already been diagnosed prior to RNA-seq based on two likely pathogenic variants in *trans*.

As described in Arriaga *et al*., this transcriptome-wide approach requires an appropriate number of controls. FRASER, the outlier detection tool used here, requires at least 25 samples of the same tissue type to generate an output model. At a cohort size of 25, an excess of intron retention events in MIGs is a reliable signature of minor spliceopathies, such as RNU4ATAC-opathy; however, specificity improves substantially with cohorts of 200 or more samples. For this reason, we recommend combining cohorts to reach enough controls. However, as Arriaga et al. found the number of outlier junctions per individual to be influenced by factors such as batch and age, we recommend applying batch correction prior to splicing outlier detection. Additionally, special care must be taken when combining cohorts such as GTEx, in which there are significant transcriptomic differences between blood collected pre and postmortem in addition to methodologic differences in RNA-seq library preparation and sequencing. It is notable, however, that the transcriptome-wide signature of RNU4ATAC-opathy in our whole blood cohort was sensitive and specific even though this cohort included samples from three sequencing centers and four protocols.

The transcriptome-wide signature described here can be replicated, although with reduced specificity, using FRASER2—an updated model of FRASER that outputs only one metric: the Jaccard index (Figure 4B). In contrast, the other main outlier detection metric, LeafCutterMD, cannot detect intron retention events, and thus cannot identify the transcriptional pattern of RNU4ATAC-opathy. We therefore recommend using intron retention events in FRASER to detect individuals with RNU4atac-opathy.

RNA-seq–to-phenotype correlations remain challenging and represent an important direction for future research work. This includes determining whether correlations can be established between genes affected by minor intron retention and the phenotypic spectrum observed in RNU4ATAC-opathy.

In conclusion, we present a cohort of 60 individuals with RNU4ATAC-opathy, highlighting novel variants, RNA sequencing findings, phenotypic variability, underreported features, and management considerations. The marked clinical overlap across phenotypic subcategories supports the use of the umbrella term “RNU4ATAC-opathy.” Given the critical role of non-coding RNA genes in human disease, we strongly advocate that both clinical and research laboratories ensure *RNU4ATAC* and other noncoding genes are included in their analysis pipelines, as diagnoses may be missed—either because the gene is not analyzed or because the broad phenotypic variability makes them difficult to recognize without a genotype-first approach. Classifying variants in *RNU4ATAC* remains challenging. We demonstrate here that RNASeq can help classify these variants by providing a functional readout, but there is an urgent need for more accessible computational approaches to predict pathogenicity in genes encoding small functional RNAs. Finally, we recommend that all individuals with a confirmed or suspected RNU4ATAC-opathy undergo comprehensive screening for associated systems, regardless of the clinical subcategory assigned.

## Supplementary Material

Supplementary Materials

## Data Availability

All the variants in *RNU4ATAC* identified in this study were submitted to ClinVar (https://www.ncbi.nlm.nih.gov/clinvar/) (GenBank: NR_023343.1) (Submitter ID 506627, Broad Rare Disease Group). The vast majority of RNA sequencing samples analyzed in this study are available in the GREGoR Consortium R03 data (https://gregorconsortium.org/data).
