# Supplementary material for: RNU4ATAC-opathy: Clinical, molecular and transcriptomic insights from a large cohort
Source: Genet Med. Author manuscript; Available in PMC 2026 Sep 11. (PMC7619276; doi:10.1016/j.gim.2026.102633)
Supplement: Supplementary file 1 [file EMS216054-supplement-Supplementary_Materials.pdf]

through the following grants, as part of the Undiagnosed Diseases Network: U01HG010218, U01HG010230, U01HG007674. The content is solely the responsibility of the author and does not necessarily represent the official views of the NIH.

**Author contributions**

Project conceptualization: D.R.M., G.L, A.O.L., F.A., J.K.; Ethics approval: D.R.M, D.B.; Clinical data contribution: D.R.M., G.L, A.D., M.E.H., T.C., M.W., L.P., M.O., A.D.N., J.K., D.W., C.S., E.D.L., S.K., S.M., L.A., O.A., H.E.S., S.A., Z.A.A., N.S., F.A., A.J., A.O.L., I.C., N.R., A.M.R.B., D.E.M., A.C.K. M.T., S.A.B., N.L.V., K.L.G., T.N.K., E.E.D., E.A., J.C., B.C., A.C., D.D.S., E.Fa., E.Fl., M.H., T.H., W.T.K., W.L., C.L., F.M., S.N., L.B.O, L.C., G.M.E., B.P.C., K.T.; Clinical data analysis: D.R.M., G.L., A.D., N.R., I.C.; RNA-seq data analysis: T.M.A., V.S.G., H.R.M.; Variant classification: K.R., M.S.B., G.L., N.R, A.J.; Figure generation: D.R.M., A.D., G.L., T.M.A, N.R., V.S.G., I.C., A.W.W.; Writing – original draft: G.L., D.R.M., A.D., T.M.A., N.R., V.S.G.; Writing – review and editing: all authors.

**Ethics declaration**

Ethical approval for the study was obtained from all participating institutions, including the Stanford University IRB (protocol #60837), MassGeneralBrigham (protocol 2016P001422), Nemours Children's Hospital (protocol #83142), the Leeds (East) Research Ethics Committee (10/H1307/2; IRAS 62971), and the Multi-centre Research Ethics Committee for Scotland (05/MRE00/74). Informed consent was obtained from all participants or their legal guardians. Consent for publication of clinical data and photographs was obtained under approved protocols at the National Human Genome Research Institute, NIH (protocol 15-HG-0130), Stanford University (protocol #60837), and Nemours (protocol #83142).

**Conflict of Interest**

D.E.M is on a scientific advisory board at Basis Genetics, is a consultant for MyOme, and holds stock options at MyOme and Basis Genetics. A.W.W. is an employee of ORIC Pharmaceuticals. K.B and F.S.A are employees of Lifera Omics. ORIC and Lifera have no involvement in the design, conduct, or publication of this research. B.P.C receives funds from Inozyme Pharma (now BioMarin) for clinical trials unrelated to RNU4ATAC. Neither Inozyme nor BioMarin had any involvement.

**Additional information (Supplemental files)**

Supplemental Table 1: Summary of phenotype
Supplemental Table 2: Variant details and classification in affected individuals
Supplemental Figure 1: Growth parameters by affected RNU4ATAC nucleotide
Supplemental Figure 2: Transcriptome-wide intron retention events in minor intron-containing genes from rare disease cohorts of muscle and whole blood RNA-seq from the Broad GREGoR Consortium site

Supplemental Figures 1 and 2

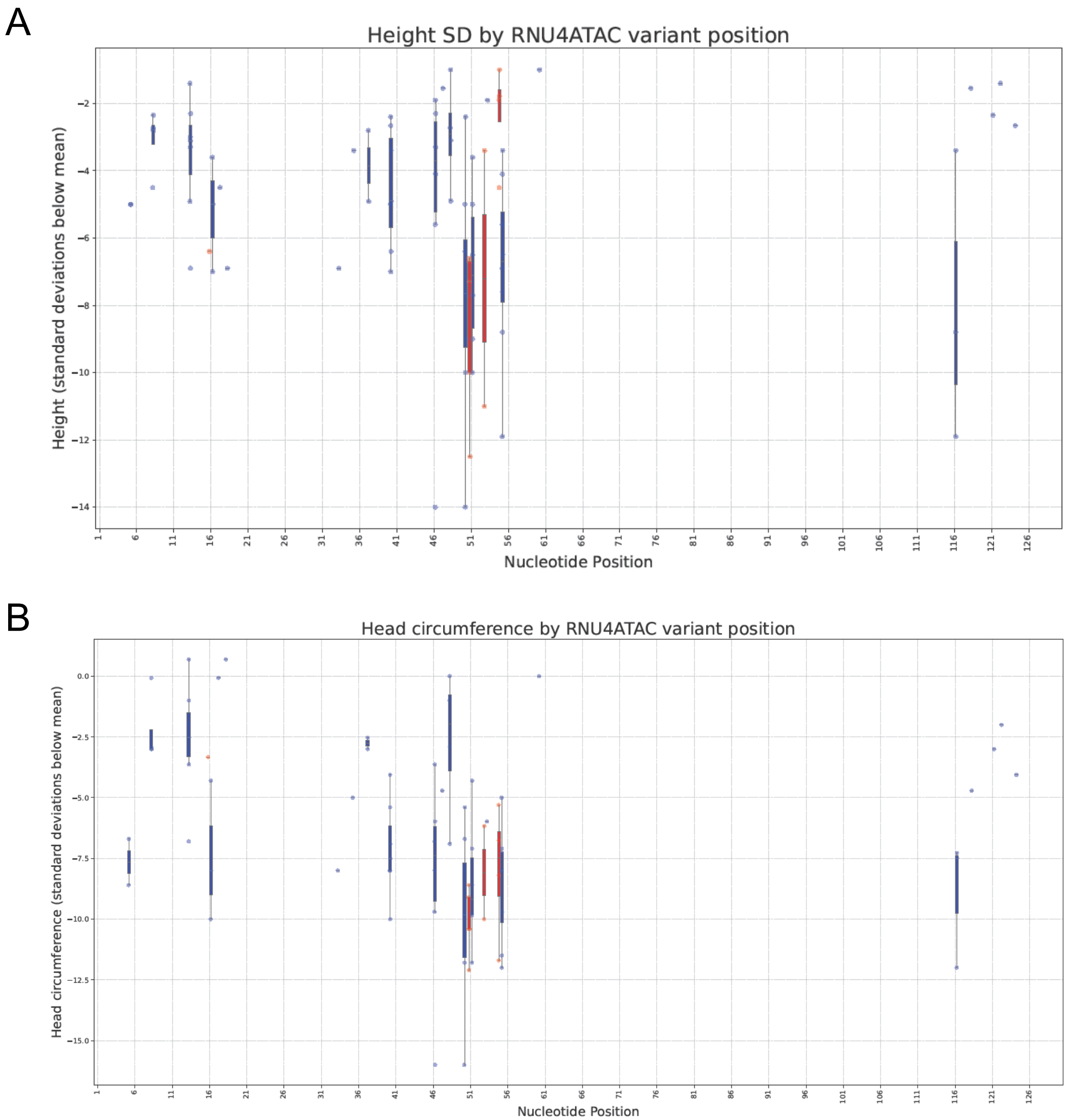


**Supplemental Figure 1: Growth parameters by affected RNU4ATAC nucleotide.** Red dots indicate individuals homozygous for a variant at that position. Blue dots indicate heterozygous variants (thus two datapoints shown per individual). Where applicable, boxplots display interquartile range with line at median value, with growth expressed as standard deviations below age-corrected population mean. Variants in the 5’ stem-loop, particularly at position n.51, have a particularly severe effect on growth. Individuals with homozygous n.51 variants had an average height of -8.9 SD, compared to -5.2 SD for the overall cohort. Their average head circumference was -10.3 SD, versus -6.8 SD in the cohort overall. Heterozygous variants around the 5’ Stem Loop were also associated with more severe growth restriction, suggesting a genotype-phenotype correlation for growth parameters. **A:** Height **B:** Head circumference.

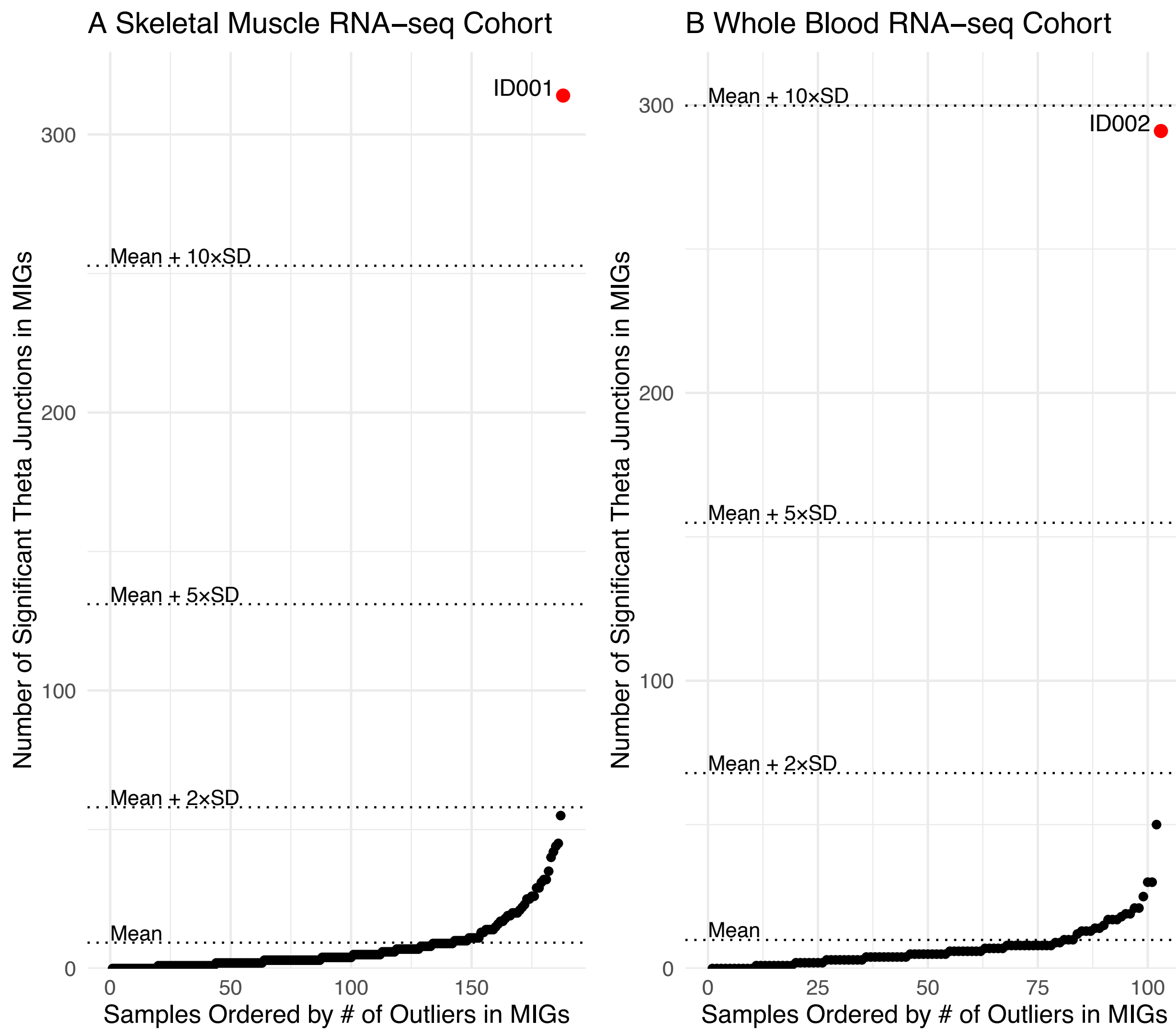


**Supplemental Figure 2: Transcriptome-wide intron retention events in minor intron-containing genes from rare disease cohorts of muscle and whole blood RNA-seq from the Broad GREGoR Consortium site**. **A.** Plot showing the number of significant ($q < 0.05$, abs($\Delta\Psi$) > 0.3) intron retention ($\theta$) events in minor intron containing genes (MIGs) from a 190 muscle RNA-seq samples, sorted by the number of significant outliers. Individual ID001 highlighted in red as an outlier within the cohort. Dotted lines indicate the mean number of outliers per sample plus multiples of the standard deviation. **B.** Same plot from a cohort of 104 whole blood RNA-seq samples. Individual ID002 highlighted in red as an outlier within the cohort.

**Consortia**

Members of the **Undiagnosed Diseases Network**: Alyssa A. Tran, Arjun Tarakad, Ashok Balasubramanyam, Brendan H. Lee, Carlos A. Bacino, Daryl A. Scott, Elaine Seto, Gary D. Clark, Hongzheng Dai, Hsiao-Tuan Chao, Ivan Chinn, James P. Orengo, Jennifer E. Posey, Jill A. Rosenfeld, Kim Worley, Lindsay C. Burrage, Lisa T. Emrick, Lorraine Potocki, Monika Weisz Hubshman, Richard A. Lewis, Ronit Marom, Seema R. Lalani, Shamika Ketkar, Tiphanie P. Vogel, William J. Craigen, Lauren Blieden, Jared Sninsky, Hugo J. Bellen, Michael F. Wangler, Oguz Kanca, Shinya Yamamoto, Christine M. Eng, Patricia A. Ward, Pengfei Liu, Adeline Vanderver, Cara Skraban, Edward Behrens, Gonench Kilich, Kathleen Sullivan, Kelly Hassey, Ramakrishnan Rajagopalan, Rebecca Ganetzky, Vishnu Cuddapah, Anna Raper, Daniel J. Rader, Giorgio Sirugo, Vaidehi Jobanputra, Allyn McConkie-Rosell, Kelly Schoch, Mohamad Mikati, Nicole M. Walley, Rebecca C. Spillmann, Vandana Shashi, Alan H. Beggs, Calum A. MacRae, David A. Sweetser, Deepak A. Rao, Edwin K. Silverman, Elizabeth L. Fieg, Frances High, Gerard T. Berry, Ingrid A. Holm, J. Carl Pallais, Joan M. Stoler, Joseph Loscalzo, Lance H. Rodan, Laurel A. Cobban, Lauren C. Briere, Matthew Coggins, Melissa Walker, Richard L. Maas, Susan Korrick, Jessica Douglas, Audrey Stephannie C. Maghiro, Cecilia Esteves, Emily Glanton, Isaac S. Kohane, Kimberly LeBlanc, Rachel Mahoney, Shamil R. Sunyaev, Shilpa N. Kobren, Brett H. Graham, Erin Conboy, Francesco Vetrini, Kayla M. Treat, Khurram Liaqat, Lili Mantcheva, Stephanie M. Ware, Breanna Mitchell, Brendan C. Lanpher, Devin Oglesbee, Eric Klee, Filippo Pinto e Vairo, Ian R. Lanza, Kahlen Darr, Lindsay Mulvihill, Lisa Schimmenti, Queenie Tan, Surendra Dasari, Adriana Rebelo, Carson A. Smith, Deborah Barbouth, Guney Bademci, Joanna M. Gonzalez, Kumarie Latchman, LéShon Peart, Mustafa Tekin, Nicholas Borja, Stephan Zuchner, Stephanie Bivona, Willa Thorson, Herman Taylor, Andrea Gropman, Barbara N. Pusey Swerdzewski, Camilo Toro, Colleen E. Wahl, Donna Novacic, Ellen F. Macnamara, John J. Mulvihill, Maria T. Acosta, Precilla D'Souza, Valerie V. Maduro, Ben Afzali, Ben Solomon, Cynthia J. Tifft, David R. Adams, Elizabeth A. Burke, Francis Rossignol, Heidi Wood, Jiayu Fu, Joie Davis, Leoyklang Petcharet, Lynne A. Wolfe, Margaret Delgado, Marie Morimoto, Marla Sabaii, MayChristine V. Malicdan, Neil Hanchard, Orpa Jean-Marie, Wendy Introne, William A. Gahl, Yan Huang, Aimee Allworth, Andrew Stergachis, Danny Miller, Elizabeth Blue, Elizabeth Rosenthal, Elsa Balton, Emily Shelkowitz, Eric Allenspach, Fuki M. Hisama, Gail P. Jarvik, Ghayda Mirzaa, Ian Glass, Kathleen A. Leppig, Katrina Dipple, Mark Wener, Martha Horike-Pyne, Michael Bamshad, Peter Byers, Sam Sheppeard, Sirisak Chanprasert, Virginia Sybert, Wendy Raskind, Nitsuh K. Dargie, Beth A. Martin, Chloe M. Reuter, Devon Bonner, Elijah Kravets, Holly K. Tabor, Jacinda B. Sampson, Jason Hom, Jennefer N. Kohler, Jonathan A. Bernstein, Kevin S. Smith, Matthew T. Wheeler, Meghan C. Halley, Page C. Goddard, Paul G. Fisher, Rachel A. Ungar, Raquel L. Alvarez, Shruti Marwaha, Terra R. Coakley, Euan A. Ashley, Ali Al-Beshri, Anna Hurst, Bruce Korf, Kaitlin Callaway, Martin Rodriguez, Tammi Skelton, Andrew B. Crouse, Jordan Whitlock, Mariko Nakano-Okuno, Matthew Might, William E. Byrd, Changrui Xiao, Eric Vilain, Jose Abdenur, Kathyrn Singh, Rebekah Barrick, Sanaz Attaripour, Suzanne Sandmeyer, Tahseen Mozaffar, Albert R. La Spada, Elizabeth C. Chao, Maija-Rikka Steenari, Alden Huang, Brent L. Fogel, Esteban C. Dell'Angelica, George Carvalho, Julian A. Martínez-Agosto, Manish J. Butte, Martin G. Martin, Naghmeh Dorrani, Neil H. Parker, Rosario I. Corona, Stanley F. Nelson, Yigit Karasozen, Aaron Quinlan, Alistair Ward, Ashley Andrews, Corrine K. Welt, Dave Viskochil, Erin E. Baldwin, John Carey, Justin Alvey, Laura Pace, Lorenzo Botto, Nicola Longo, Paolo Moretti, Rebecca Overbury, Russell Butterfield, Steven Boyden, Thomas J. Nicholas, Matt Velinder, Gabor Marth, Pinar Bayrak-Toydemir, Rong Mao, Monte Westerfield, Brian Corner, John A. Phillips III, Kimberly Ezell, Lynette Rives, Rizwan Hamid, Serena Neumann, Ashley McMinn, Joy D. Cogan, Thomas Cassini, Alex Paul, Dana Kiley, Daniel Wegner, Erin McRoy, Jennifer Wambach, Kathy Sisco, Patricia Dickson, F. Sessions Cole, Dustin Baldridge, Jimann Shin, Lilianna Solnica-Krezel, Stephen Pak, Timothy Schedl, Hector Rodrigo Mendez, Brianna

Tucker, Beatriz Anguiano, Mia Levanto, Suha Bachir, Laurens Wiel, Stephen B Montgomery, Tanner D Jensen, John E. Gorzynski, Sara Emami, Laura Keehan, Jennifer Schymick, Taylor Maurer, Alexander Miller, Andres Vargas, Amanda M. Shrewsbury, Bianca E. Russell, Layal F. Abi Farraj, Elizabeth A Worthey, Tarun KK Mamidi, Brandon M Wilk, Rachel Li, Jennifer Morgan, Chun-Hung Chan, Paul Berger, Mohamad Saifeddine, Isum Ward, Jason Schend, Megan Bell, Francisco B Velasquez, Taylor Beagle, Miranda Leitheiser, Runjun Kumar , Donald Basel, Michael Muriello, Brett Bordini, Michael Zimmermann, Abdul Elkadri, James Verbsky, Julie McCarrier.

Members of the **Genomics Research to Elucidate the Genetics of Rare Diseases** (GREGoR) consortium: Siwaar Abouhala, Kaileigh Ahlquist, Miguel Almalvez, Emily Alsentzer, Raquel Alvarez, Mutaz Amin, Kailyn Anderson, Peter E. Anderson, Euan Ashley, Themistocles Assimes, Light Auriga, Christina Austin-Tse, Michael J. Bamshad, Rebekah Barrick, Rebekah Barrick, Samantha Baxter, Sairam Behera, Shaghayegh Beheshti, Gill Bejerano, Sami Belhadj, Seth Berger, Jon Bernstein, Sabrina Best, Benjamin Blankenmeister, Elizabeth E. Blue, Krista Bluske, Eric Boerwinkle, Emily Bonkowski, Devon Bonner, Philip M. Boone, Philip M. Boone, Leandros Boukas, Denver Bradley, Harrison Brand, Kati J. Buckingham, Daniel Calame, Colleen Carlston, Jennefer Carter, Silvia Casadei, Lisa Chadwick, Clarisa Chavez, Ziwei Chen, Yong-Han Cheng, Ivan Chinn, Jessica X. Chong, Zeynep Coban-Akdemir, Andrea J. Cohen, Sarah Conner, Matthew P. Conomos, Karen Coveler, Ya Allen Cui, Zain Dardas, Colleen P. Davis, Moez Dawood, Ivan de Dios, Celine de Esch, Celine de Esch, Emmanuèle Délot, Salil Deshpande, Stephanie DiTroia, Harsha Doddapaneni, Haowei Du, Michael Duyzend, Michael Duyzend, Michael Duyzend, Iman Egab, Evan E. Eichler, Sara Emami, Ivy Evergreen, Jamie Fraser, Vincent Fusaro, Mira Gandhi, Vijay Ganesh, Brandon Garcia, Kiran Garimella, Richard Gibbs, Sophia B. Gibson, Casey Gifford, Carmen Glaze, Pagé Goddard, Stephanie Gogarten, Nikhita Gogate, William W. Gordon, John E. Gorzynski, William Greenleaf, Christopher Grochowski, Emily Groopman, Emily Groopman, Rodrigo Guarischi-Sousa, Sanna Gudmundsson, Jonas (A. Gus) Gustafson, Stacey Hall, Caitlin Harrington, John Harting, William T. Harvey, Sohaib Hassan, Megan Hawley, Benjamin D. Heavner, Martha Horike-Pyne, Yun-Hua Hsiao, Jianhong Hu, Yongqing Huang, Karan Jaisingh, Minal Jamsandekar, Gail P. Jarvik, Tanner Jensen, Shalini Jhangiani, David Jimenez-Morales, Christopher Jin, Aimee Juan, Ahmed K. Saad, Jessica Kain, Rachid Karam, Laura Keehan, Rupesh Kesharwani, Charles Hadley King, Julia Klugherz, Arthur Ko, Anshul Kundaje, Soumya Kundu, Samuel M. Lancaster, Katie Larsson, Arthur Lee, Gabrielle Lemire, Jesse Levine, Richard Lewis, Wei Li, Yidan Li, Pengfei Liu, Bojan Losic, Jonathan LoTempio, James (Jim) Lupski, Jialan Ma, Daniel MacArthur, Annelise Y. Mah-Som, Medhat Mahmoud, Brian Mangilog, Dana Marafi, Sofia Marmolejos, Daniel Marten, Eva Martinez, Colby T. Marvin, Shruti Marwaha, F. Kumara Mastrorosa, Dena Matalon, Taylor Maurer, Susanne May, Sean R. McGee, Lauren Meador, Heather C. Mefford, Hector Rodrigo Mendez, Olfa Messaoud, Alexander Miller, Danny E. Miller, Stephen Montgomery, Yulia Mostovoy, Mariana Moyses, Mariana Moyses, Chloe Munderloh, Donna Muzny, Ashana Neale, Sarah C. Nelson, Matthew B. Neu, Thuy-mi P. Nguyen, Jonathan Nguyen, Robert Nussbaum, Emily O'Heir, Melanie O'Leary, Briana O'Leary, Sebastian Ochoa Gonzalez, Jeren Olsen, Ikeoluwa Osei-Owusu, Anne O‚ÄôDonnell-Luria, Miranda P.G. Zalusky, Evin Padhi, Lynn Pais, Piyush Panchal, Shruti Pande, Karynne E. Patterson, Sheryl Payne, Davut Pehlivan, Davut Pehlivan, Paul Petrowski, Alicia Pham, Georgia Pitsava, Astaria/Sara Podesta, Elizabeth Porter, Jennifer Posey, Jaime Prosser, Guanghao Qi, Wanqiong Qiao, Thomas Quertermous, Archana Rai, Heidi Rehm, Chloe Reuter, Matthew A. Richardson, Andres Rivera-Munoz, Lindsay Romo, Oriane Rubio, Kathryn Russell, Aniko Sabo, Monica Salani, Monica Salani, Kaitlin Samocha, Alba Sanchis-Juan, Sarah Savage, Stuart Scott, Evette Scott, Adriana E. Sedeño-Cortés, Fritz Sedlazeck, Jillian Serrano, Gulalai Shah, Ali Shojaie, Moriel Singer-Berk, Mugdha Singh, Riya Sinha, Joshua D Smith, Kevin Smith, Hana Snow,

Michael Snyder, Kayla Socarras, Olivia M. Sommerland, Lea M. Starita, Brigitte Stark, Sarah Stenton, Sarah Stenton, Andrew B. Stergachis, Adrienne Stilp, Suchitra Sudarshan, V. Reid Sutton, V. Reid Sutton, Jui-Cheng Tai, Jui-Cheng Tai, Michael Talkowski, Christina Tise, Catherine C. Tong, Philip Tsao, Rachel Ungar, Grace VanNoy, Eric Vilain, Gaby Villard, Isabella Voutos, Kim Walker, Juliana Walrod, Chia-Lin Wei, Ben Weisburd, Jeffrey M Weiss, Chris Wellington, Ziming Weng, Lauren Westerfield, Emily Westheimer, Marsha Wheeler, Matthew Wheeler, Laurens Wiel, Michael Wilson, Monica Wojcik, Issac Wong, Frank Wong, Quenna Wong, Changrui Xiao, Jiaoyang Xu, Rachita Yadav, Rachita Yadav, Yao Yang, Qian Yi, Jiye Yu, Bo Yuan, Christina Zakarian, Jianhua Zhao, Jimmy Zhen

Supplemental Table 1

Click here to access/download
**Large Excel File**
Supplemental_table_1.xlsx

Supplemental Table 2

Click here to access/download
**Large Excel File**
Supplemental_Table_2_rev.xlsx
